# Clinical evaluation of pregnant women with SARS-COV2 pneumonia: a real-life study from Egypt

**DOI:** 10.1186/s42506-021-00092-z

**Published:** 2021-11-04

**Authors:** Samy Zaky, Hossam Hosny, Gehan Elassal, Noha Asem, Amin Abdel Baki, Ehab Kamal, Akram Abdelbary, Ahmad Said, Hamdy Ibrahim, Khaled Taema, Wagdy Amin, Sherief Abd-Elsalam, Shaimaa Soliman, Hend Salah Abdelmenam, Ahmed S. Mohamed, Mohamed Elnady, Mohamed Hassany, Hala Zaid

**Affiliations:** 1 Hepatogastroenterology and Infectious Diseases Department, Faculty of Medicine, Al-Azhar University, Cairo, Egypt; 2 Chest Diseases Department, Head of Pulmonary Hypertension Unit, Faculty of Medicine, Cairo University, Cairo, Egypt; 3 Pulmonary Medicine Department, Faculty of Medicine, Ain Shams University, Cairo, Egypt; 4 Public Health Department, Faculty of Medicine, Cairo University, Cairo, Egypt; 5 National Hepatology and Tropical Medicine Research Institute (NHTMRI), Cairo, Egypt; 6 Tropical Medicine Department. Medical Division, National Research Center, Cairo, Egypt; 7 Critical Care Medicine Department, Faculty of Medicine, Cairo University, Cairo, Egypt; 8 Fever Hospitals, Ministry of Health and Population (MOHP), Cairo, Egypt; 9 MOHP, Cairo, Egypt; 10 Tropical Medicine and Infectious Diseases Department, Faculty of Medicine, Tanta University, Tanta, Egypt; 11 Public Health and Community Medicine Department, Menoufia University, Menoufia, Egypt; 12 Obstetrics and Gynecology Department, Al Zahraa University Hospital, Cairo, Egypt; 13 Chest Diseases Department, Faculty of Medicine, Tanta University, Tanta, Egypt; 14 Pulmonology Department, Faculty of Medicine, Cairo University, Cairo, Egypt

**Keywords:** COVID-19, Pregnancy, Outcomes, Mortality, Egypt, Ventilation

## Abstract

**Background:**

Knowledge about the outcome of COVID-19 on pregnant women is so important. The published literature on the outcomes of pregnant women with COVID-19 is confusing. The aim of this study was to report our clinical experience about the effect of COVID-19 on pregnant women and to determine whether it was associated with increased mortality or an increase in the need for mechanical ventilation in this special category of patients.

**Methods:**

This was a cohort study from some isolation hospitals of the Ministry of Health and Population, in eleven governorates, Egypt. The clinical data from the first 64 pregnant women with COVID-19 whose care was managed at some of the Egyptian hospitals from 14 March to 14 June 2020 as well as 114 non-pregnant women with COVID-19 was reviewed.

**Results:**

The two groups did not show any significant difference regarding the main outcomes of the disease. Two cases in each group needed mechanical ventilation (*p* 0.617). Three cases (4.7%) died among the pregnant women and two (1.8%) died among the non-pregnant women (*p* 0.352).

**Conclusions:**

The main clinical outcomes of COVID-19 were not different between pregnant and non-pregnant women with COVID-19. Based on our findings, pregnancy did not exacerbate the course or mortality of COVID-19 pneumonia.

## Introduction

The virus that causes COVID-19 and the virus that caused the severe acute respiratory syndrome (SARS) outbreak in 2003 are genetically related to each other, but the diseases caused by them are completely different. SARS was more deadly but much less contagious than COVID-19 [1, 2]. COVID-19 is clinically milder than MERS or SARS in terms of severity and mortality (the death rate for COVID-19 appears to be around 2–5% [1–4].

As cases of COVID-19 continue to rise in countries across the region, health systems face tremendous pressure to manage COVID-19 patients [1–8].

The published literature on the outcomes of pregnant women with COVID-19 is very confusing [6–17]. As of 20 April 2020, 51 papers have reported primary data on COVID-19 during pregnancy [6–32]. However, there were a lot of problems with these information sources. First, there was double counting of cases. This was a particular problem with early reports from China, and the authors rarely explained it. Second, most of the reports were of very small numbers; meanwhile; the larger studies reported few details. Another problem was that one third of these papers were individual case reports, which were likely biased toward severe or conditions without classical presentation.

Pregnant women are particularly vulnerable to respiratory pathogens and acute pneumonia, because they are in an immunosuppressed state, and additionally the adaptive physiological changes during pregnancy make them intolerant to hypoxia [25, 26]. The aim of this study is to clinically evaluate the effect of COVID-19 on pregnant women and to determine whether it was associated with increased mortality or need for mechanical ventilation in this special category of patients.

## Methods

### Study design and settings

This was a cohort study from some isolation hospitals affiliated to the Ministry of Health and Population in 11 governorates in Egypt. We reviewed clinical data from the first 64 pregnant women with COVID-19 whose care was managed at these Egyptian hospitals from 14 March to 14 June 2020 as well as 114 non-pregnant women with COVID-19.

### Data collection methods

All pregnant females were subjected to full history taking, clinical examination, and full-laboratory data.

The main outcomes were the difference in mortality rates and the need for mechanical ventilation.

The research was approved by the Ministry of Health and Population Research Ethics Committee. An informed consent was obtained from all participants in this research. Privacy of participants and confidentially of the data were assured. Risks and Benefits were declared.

### Statistical analysis

Data were expressed in number (No.), percentage (%) mean ($\overline{\mathrm{x}}$), and standard deviation (SD). Student’s *t* test was used for comparison of quantitative normally distributed variables between two groups, while Mann–Whitney’s test was used for not normally distributed ones. Chi-square test (*χ*2) was used to study association between qualitative variables. Whenever any of the expected cells were less than five, Fisher’s exact test was used. A univariate logistic regression analysis was performed to ascertain the effects of possible risk factors on the final outcome. Two-sided *P* value of < 0.05 was considered statistically significant.

## Results

The study included 64 pregnant women with mean gestational age of 10.73 ± 2.15 weeks. The mean age of the pregnant women was 34.93 ± 6.27 years with no significant difference from the non-pregnant group (*p* = 0.109). Among the pregnant COVID-19 patients, 9 (14.1) were in the first, 16 (25.0) in the second and 39 (60.9) in the third trimester. Four (6.25%) of the pregnant group had hypertension, 3 (4.7%) had diabetes mellitus and 4 (6.25%) had other comorbidities including cancer, asthma, and rheumatic heart disease. There was no significant difference between the 2 groups regarding different comorbidities, percentage of oxygen saturation at presentation or disease severity (*p* = 0.700, 0.206, and 0.363 respectively) (Table 1).

**Table 1:** Comparison between the pregnant COVID-19 and non-pregnant COVID-19 patients regarding their clinical characteristics, Egypt, 2020

Variable	Pregnant COVID-19 (*n* = 64)	Non-pregnant COVID-19 (*n* = 114)	*P* value
**Age (in years)**	34.93 ± 6.27	36.91 ± 8.60	0.109
**Trimester**			
First	9 (14.1)	**–**	**–**
Second	16 (25.0)		
Third	39 (60.9)		
**Co-morbidities**			
No	53 (82.8)	88 (77.2)	0.700
HTN	4 (6.25)	11 (9.6)	
DM	3 (4.7)	9 (7.9)	
Others	4 (6.25)	6 (5.3)	
**O2 saturation**			
100–95%	50 (78.1)	74 (64.9)	0.206
95–90%	6 (9.4)	23 (20.2)	
90–85%	5 (7.8)	8 (7.0)	
< 85%	3 (4.7)	9 (7.9)	
**Disease severity**			
Mild	44 (68.8)	75 (65.8)	
Moderate	16 (25.0)	36 (31.6)	0.363
Severe	4 (6.2)	3 (2.6)	

*HTN* hypertension, *DM* diabetes mellitus

The laboratory investigation did not show any significant difference between the two groups (Table 2).

**Table 2:** Comparison between the pregnant COVID-19 and non-pregnant COVID-19 patients regarding their laboratory characteristics, Egypt, 2020

Variable	Pregnant COVID (*n* = 64) Mean ± SD	Non-pregnant COVID (*n* = 114) Mean ± SD	*P* value
**Hb**	10.73 ± 2.15	11.23 ± 1.54	0.125
**WBCs**	7.34 ± 3.11	6.59 ± 3.01	0.116
**Lymphocytes**	11.13 ± 14.65	11.94 ± 9.32	0.653
**Neutrophils**	76.25 ± 152.69	74.89 ± 139.34	0.951
**Platelets**	245.42 ± 114.78	238.17 ± 121.0	0.696
**CRP**	16.67 ± 27.06	24.15 ± 31.52	0.085
**ALT**	26.25 ± 53.51	29.60 ± 49.23	0.673
**AST**	29.08 ± 43.88	28.00 ± 14.66	0.131
**Creatinine**	0.68 ± 0.46	0.82 ± 1.09	0.664
**Albumin**	3.17 ± 6.87	3.24 ± 5.18	0.938
**D dimer**	21.06 ± 117.49	46.42 ± 241.15	0.285
**Serum ferritin**	320.85 ± 223.32	360.78 ± 509.82	0.186

The two groups did not show any significant difference regarding the main outcomes of the disease. Two cases in each group needed mechanical ventilation (*p* = 0.617). Three cases (4.7%) died among the pregnant women and two (1.8%) died among the non-pregnant women (*p* = 0.352), (Table 3)

**Table 3:** Comparison between the pregnant COVID-19 and non-pregnant COVID-19 patients regarding the main clinical disease-outcomes, Egypt, 2020

Variable	Pregnant COVID (*n* = 64) no. (%)	Non-pregnant COVID (*n* = 114) no. (%)	*P* value
**Need for mechanical ventilation**	2 (3.2)	2 (1.8)	0.617
**Mortality**			
Died	3 (4.7)	2 (1.8)	0.352
Survived	61 (95.3)	112 (98.2)	

The univariate analysis showed that none of the possible risk factors was associated with the mortality among the studied patients including pregnancy (Table 4).

**Table 4:** Univariate analysis of possible risk factors associated with patients’ mortality in the studied groups, Egypt, 2020

Factor	Univariate analysis			
	*P* value	OR	95% CI	
			Lower	Upper
**Age**	0.425	0.953	0.846	1.073
**O2 saturation**	0.779	1.174	0.383	3.600
**ALT**	0.904	1.002	0.969	1.036
**Creatinine**	0.830	1.249	0.163	9.580
**Ferritin**	0.341	1.003	0.997	1.009
**CRP**	0.074	0.977	0.953	1.002
**D dimer**	0.987	–	–	–
**Presence of comorbidity**	0.731	0.676	0.073	6.296
**Pregnancy**	0.274	0.363	0.059	2.23

No statistically significant difference was noted among patients in different trimesters regarding the disease severity (*p* = 0.552). Four cases had severe COVID-19 infection; they were all in the 3rd trimester. No severe cases were reported in the 1st or 2nd trimesters. Although all the maternal deaths occurred in the 3rd trimester, this was not statistically significant (*p* = 0.715) (Table 5).

**Table 5:** Maternofetal clinical features and outcome among different trimesters, Egypt, 2020

Clinical feature	1st trimester (*n* = 9) No. (%)	2nd trimester (*n* = 16) No (%)	3rd trimester (*n* = 39) No. (%)	*P* value
**Disease severity**				
Mild	7 (77.8)	11 (68.8)	26 (66.6)	0.552
Moderate	2 (22.2)	5 (31.2)	9 (23.1)	
Severe	0 (0.0)	0 (0.0)	4 (10.3)	
**Maternal deaths**	0 (0.0)	0 (0.0)	3 (7.7)	0.715
**Fetal outcome**				
IUFD	1 (11.1)	0 (0.0)	1 (2.6)	0.406
Hydrocephalus	0 (0.0)	1 (6.3)	0 (0.0)	
Preterm labor	0 (0.0)	1 (6.3)	0 (0.0)	

Among all the pregnant patients, two cases (3.1%) had intrauterine fetal death (IUFD), one baby was born with hydrocephalus (1.6%) and one (1.6%) was born pre-term. There was no significant association between the trimester and the fetal outcome (*p* = 0.406) (Table 5).

## Discussion

This study included 64 pregnant women with mean gestational age of 10.73 ± 2.15 weeks. There was no significant difference between the 2 groups regarding different comorbidities or percentage of oxygen saturation at presentation (*p* = 0.700 and 0.206 respectively). So, both groups were not significantly different regarding the baseline demographic criteria or regarding the presence of comorbid diseases.

Interestingly, the two groups did not show any significant difference regarding the main outcomes of the disease. These results indicate that the main clinical outcomes of COVID-19 were not different between pregnant and non-pregnant women with COVID-19.

The National Institute of Health (NIH) (2020) reported that surveillance data released by the CDC in June 2020 showed that COVID-19-related death rates were similar in the pregnant and non-pregnant populations. Pregnancy outcomes such as preterm birth or pregnancy loss were not evaluated [28]. This report agrees with our conclusion that the mortality risks were similar among pregnant and non-pregnant women with COVID-19 infection.

On the other side, an Iranian case series reported maternal deaths in seven pregnant women among nine pregnant women who were infected with the novel coronavirus [29]. This was extremely different from our findings. However, and generally speaking, individual case reports were likely biased toward severe or unusual disease.

### Limitations of the study

The main limitation of the study is the short time of the outcome, therefore, further studies about the long-term outcomes for the newborn and whether mother-to-child transmission are required. Nevertheless, the data in this study allow for better understanding of the clinical outcomes of COVID-19 infection in pregnancy and whether they were different between pregnant and non-pregnant females. Further studies discussing the follow-up of recovered pregnant women and the impact on their babies, type of delivery, and puerperium are highly recommended.

## Conclusions

The main clinical outcomes of COVID-19 were not different between pregnant and non-pregnant women with COVID-19. Based on our findings, pregnancy did not exacerbate the course or mortality of COVID-19 pneumonia.

## Data Availability

Data will be available from the authors on reasonable request.

## Abbreviations

Alb: Albumin
ALT: Alanine transaminase
AST: Aspartate transaminase
BMI: Body mass index
CBC: Complete blood count
COVID: Coronavirus infectious disease
CRP: C-reactive protein
DM: Diabetes mellitus
Hb: Hemoglobin
HBV: Hepatitis B virus
HCV: Hepatitis C virus
HTN: Hypertension
No: Number
PCR: Polymerase chain reaction
PLT: Platelets
SARS: Serious acute respiratory distress syndrome
SD: Standard deviation
SVR: Sustained virological response
USA: United States of America
WBCs: White blood cells
